# Reconstruction of the Ureter Using the Boari Flap and Psoas Hitch Techniques in a Patient With Damage to the Proximal Part of the Ureter: A Case Report

**DOI:** 10.7759/cureus.66522

**Published:** 2024-08-09

**Authors:** Jan T Stępka, Maciej Konopka, Michał Olszewski, Julia Woźna, Maciej Kosiński, Wiktor Szymajda, Tomasz Deja

**Affiliations:** 1 Department of Urology, Poznan University of Medical Sciences, Poznan, POL; 2 Department of Urology, Ministry of Internal Affairs Hospital Poznan, Poznan, POL

**Keywords:** kidney mobilization, proximal ureter, reconstruction, psoas hitch, boari flap

## Abstract

Although the Boari flap technique is commonly described in the literature as a method for primarily distal and middle ureteral injuries, it can also be used for reconstructing the proximal segment of the ureter. In this case study, we present a patient who underwent gynecological surgery with subsequent damage to the proximal ureter, and who underwent ureteral reconstruction using methods such as kidney mobilization, Boari flap, and psoas hitch. The postoperative period was uncomplicated, and after a six-month follow-up, the reconstructed ureter is functioning well, and the patient is in good health.

## Introduction

Ureteral injuries, which can occur during surgical procedures, often involve damage to the proximal segment of the ureter [1]. These complications most commonly occur during gynecological procedures due to the close anatomical relationship between the ureter and the female reproductive organs, which poses a significant risk during surgery [1]. Such trauma needs to be properly managed, for which reconstructive techniques are helpful - one of these is the Boari flap (BF). This ureteral reconstruction technique involves creating a flap from the bladder wall and tubularizing it to replace the damaged segment of the ureter, thereby restoring its continuity and function. This technique is primarily documented in the literature for its use in the reconstruction of the distal and middle parts of the ureter [1-7].

However, there are indications that this procedure can be used for proximal ureter reconstruction [8] as adaptability of the BF technique with addition to additional techniques such as psoas hitch [8] is particularly significant in complex surgical scenarios where alternative reconstructive options may be limited or less effective.

## Case presentation

**Figure 1:**
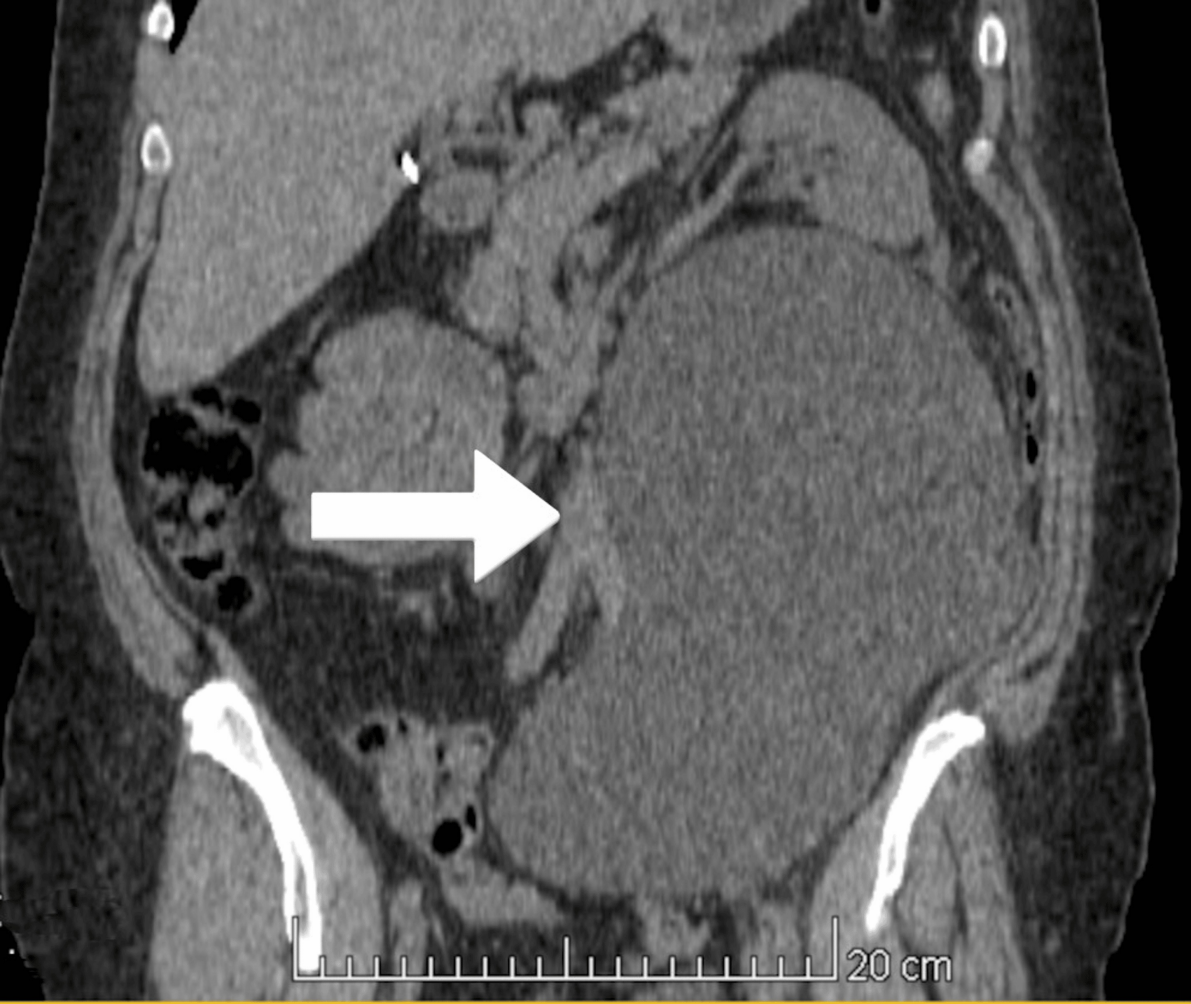
Large urinoma observed at the patient’s initial admission to the urology department, caused by a ureteral injury.

**Figure 2:**
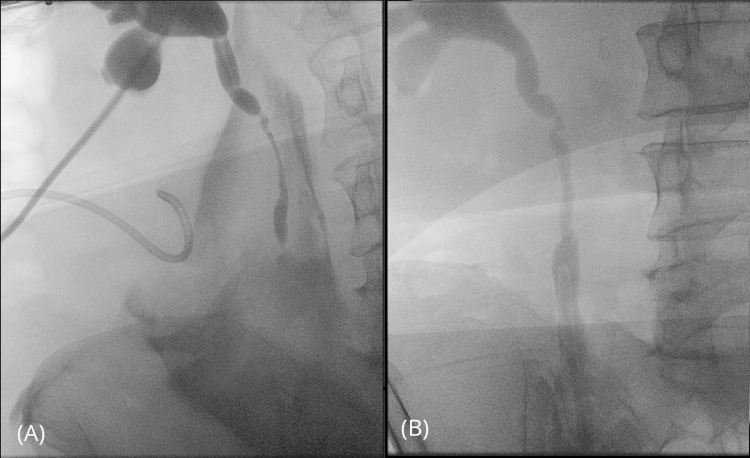
(A) Preoperative descending pyelography showing a visible ureteral stricture and a drain used for draining the urinoma. (B) Postoperative ascending pyelography displaying the reconstructed ureter, which has been anastomosed with the Boari flap (BF).

In May 2023, a 44-year-old obese patient underwent left ovarian cyst and adnexa removal surgery. In August, she was admitted to the Urology Department in MSWiA Hospital in Poznan, as she developed significant sacral pain and self-reported reduced urine output from a retroperitoneal fluid collection, diagnosed as a urinoma by a CT scan (Figure 1). In ascending and descending pyelography (Figure 2A), narrowing of the ureter was detected at a height of 8 cm distally from the ureteropelvic junction - attempts at stenting and inserting a catheter through the narrowing failed. Obstructive uropathy was diagnosed, necessitating ureteral reconstruction.

Given that the patient was three months post-surgery, during which the injury occurred, the decision to schedule delayed reconstruction [5] was adopted to allow the tissues to heal rather than immediate repair. A drain was inserted to evacuate the urinoma. The day after drainage, a temporary nephrostomy was established, and a deferred surgical procedure was planned. The patient was subsequently discharged with recommendations. The patient's kidney was notably mobile, and her high body weight contributed to the fact that the patient was readmitted several times in August-September for the reinsertion of the nephrostomy tube, which had dislodged.

**Figure 3:**
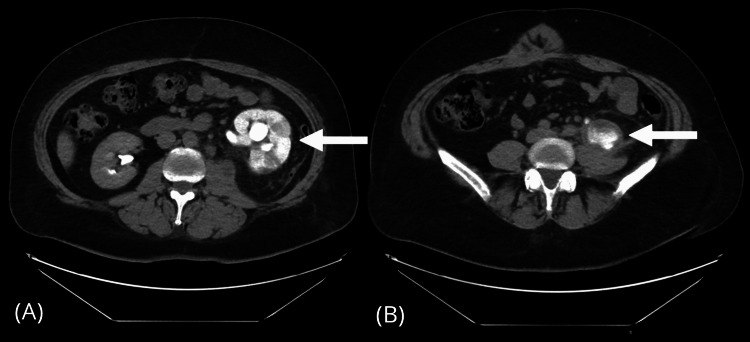
(A, B) CT scans of the patient during her last admission before surgery. (A) The left kidney showing retained contrast two hours post-administration. (B) Fluid collection on the patient’s left side indicating a urinoma with contrast. A linea alba hernia is also visible in the patient’s abdominal region.

On September 30, the patient was again urgently readmitted due to a dislocated nephrostomy catheter. Attempts at repositioning were unsuccessful. CT scans indicated diminished enhancement in the left kidney parenchyma (Figure 3A), along with a urinoma evidenced by contrast-filled fluid in the retroperitoneal space (Figure 3B). Additionally, a linea alba hernia was identified. Given these complications, it was promptly determined that the patient required urgent ureteral reconstruction.

The patient, under general anesthesia and in the dorsal lithotomy position, had her abdominal cavity opened midline from above the navel to the pubic symphysis. Intestinal adhesions were released, including those near the descending colon, and an automatic retractor was placed. Common iliac vessels were identified and below them, an encapsulated fluid collection was found and aspirated. Dissection extended to the left kidney's lower pole, where the ureter was initially unidentifiable. After opening the renal fat capsule, dissection continued medially. The ureter was eventually located on the medial side of the fluid collection's upper pole.

**Figure 4:**
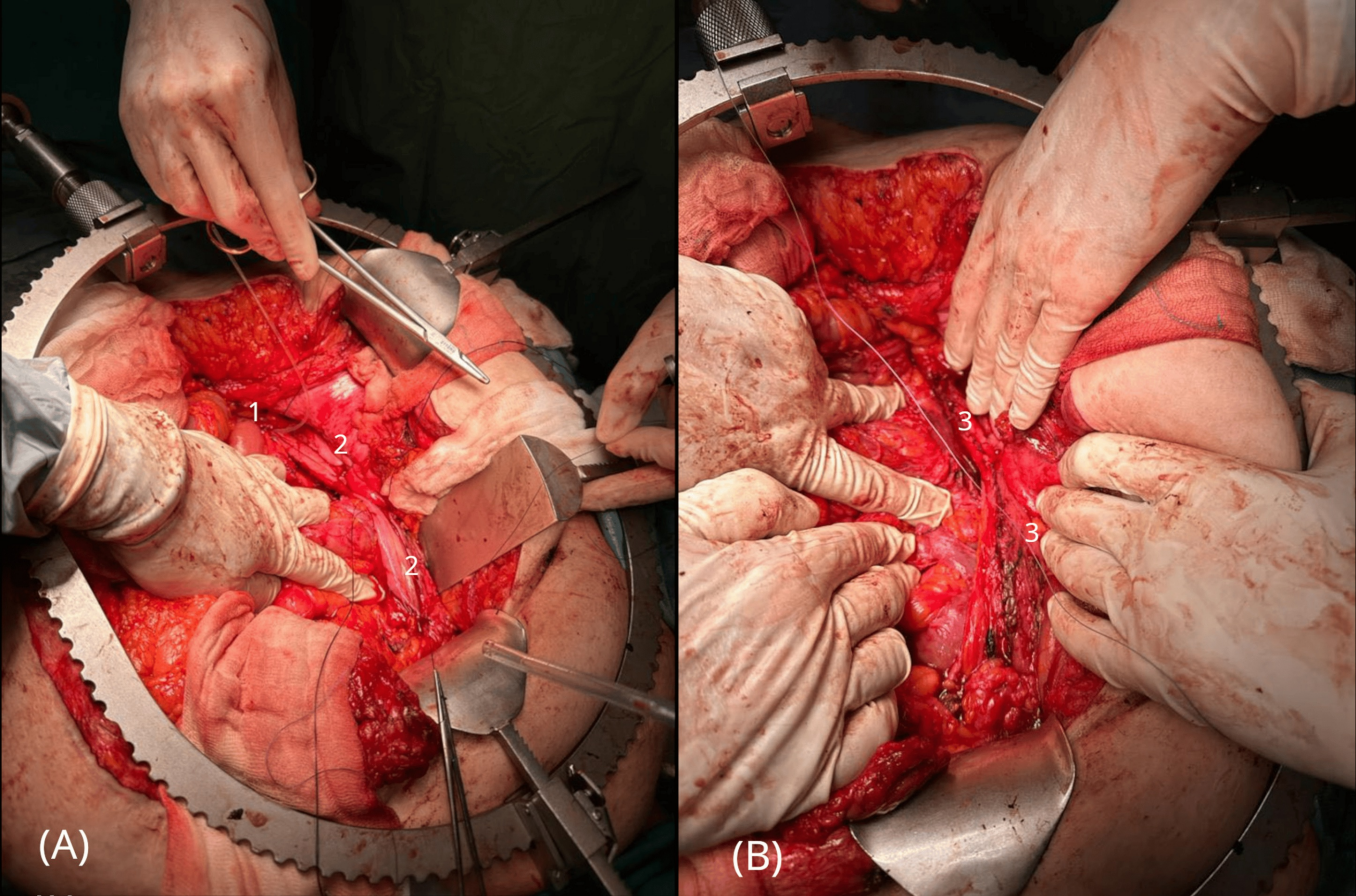
(A, B). Reconstruction surgery of the proximal ureter using the Boari flap (BF) technique. A. (1) Proximal ureter with Nelaton stent in place. (2) BF before closure. B. (3) BF after closure.

The dissected proximal ureter measured 6 cm. Ureteral spatulation was performed followed by kidney mobilization within its fat capsule. The urinary bladder was mobilized bilaterally, and its anterior wall was dissected. A bladder flap of approximately 44 mm x 110 mm was excised. A psoas hitch maneuver was performed. To create the anastomosis, the ureter was end-to-end joined with the BF at the level of the lower pole of the kidney, about 2/3 cm above the suspected place of ureter crossing with common iliac vessels (Figures 4A, 4B). Before the anastomosis, the lack of tension on the approaching tissues was confirmed.

A double J catheter and a three-way urinary bladder catheter were inserted, followed by abdominal drainage and closure with anti-evisceration and single sutures. The operation resulted in a blood loss of 300 mL and lasted six hours.

The patient had an uncomplicated ward stay, with the drain removed on day 5 post-op, and discharged home on day 7 with recommendations. The patient was advised to avoid heavy lifting and strenuous activities, maintain hydration, follow a balanced diet, and monitor for signs of infection or complications. Follow-up appointments were scheduled to monitor the healing process, and the patient was instructed on proper catheter care and to report any significant changes in urine output or severe pain. Five weeks after the surgery, ascending pyelography was performed, showing the reconstructed ureter (Figure 2B), and a double J catheter was removed. Six months follow-up later, the patient reported no complaints, and urination was normal suggesting that reconstruction was successful.

## Discussion

The BF method is mainly described in the literature as applicable to the distal or middle segment of the ureter [1-7]. However, a retrospective study comparing the use of this technique in the reconstruction of proximal ureteral damage versus middle and distal damage showed that the BF technique is reliable for ureteral stricture reconstruction regardless of location, offering similar success rates in reconstruction [8]. There are instances where the BF technique can be applied even in more advanced ureteral damage than in our case. Reports of replacing the entire ureter with a BF demonstrate the extent to which this method can be utilized with additional techniques [9,10].

Other methods available for repairing the proximal segment of the ureter include ureteroureterostomy, transureteroureterostomy, and bowel interposition graft [11]. Ureteroureterostomy is an effective method of ureteral reconstruction [11], but it was not possible due to extensive damage to the ureter in our patient. Transureteroureterostomy, also with a good prognosis [11], requires a sufficiently long ureter - we did not have enough ureter to consider this option in our case. Another method is replacing the ureter with an intestinal graft. It shows good results in ureter reconstruction and is widely used for proximal ureteral damage [11], but it is associated with complications such as obstruction, electrolyte disturbances, or short bowel syndrome [8]. This was another method we considered if issues arose with the primary choice.

Based on the aforementioned reasons, we decided to go with BF. To perform this technique, adequate bladder capacity, a wide flap, and sufficient bladder mobilization are necessary. While not a perfect method, postoperative complications such as bladder function disorders can occur [8]. The main concern with using BF in the proximal part is whether the created bladder flap will adequately reach the ureter. Methods such as psoas hitch, which involves attaching the bladder to the psoas muscle to elevate and support it, kidney mobilization, and downward nephropexy significantly reduce the distance the flap must reach, which is crucial as the created anastomosis must not be under tension [11]. Harada et al. demonstrate that mobilizing the kidney alone can gain up to 7 cm of additional ureteral length [12]. In our case, we performed such kidney mobilization, which, along with the psoas hitch, allowed the flap to reach the proximal ureter and create a tension-free anastomosis. The dimensions of the bladder flap were carefully chosen, adhering to the recommended length-to-width ratio of no more than 3:1 [11] and a base width of at least 4 cm to ensure adequate vascular supply to the flap's apex. Downward nephropexy was not used in our approach.

## Conclusions

This case underscores the importance of individualized surgical planning and the consideration of all available options. There are several methods to repair the proximal ureter. Although the BF is not the first choice for reconstruction in this region, this case report demonstrates that using BF, along with psoas hitch and kidney mobilization, is feasible and can yield positive outcomes. Our patient's case highlights the technique's potential versatility and adaptability in complex scenarios. However, it is crucial to remember that BF should be considered a secondary option when standard methods are unsuitable.

## References

[1] Pereira BM, Ogilvie MP, Gomez-Rodriguez JC (2010). A review of ureteral injuries after external trauma. Scand J Trauma Resusc Emerg Med.

[2] Symons S, Kurien A, Desai M (2009). Laparoscopic ureteral reimplantation: a single center experience and literature review. J Endourol.

[3] Gild P, Kluth LA, Vetterlein MW, Engel O, Chun FK, Fisch M (2018). Adult iatrogenic ureteral injury and stricture-incidence and treatment strategies. Asian J Urol.

[4] Wenske S, Olsson CA, Benson MC (2013). Outcomes of distal ureteral reconstruction through reimplantation with psoas hitch, Boari flap, or ureteroneocystostomy for benign or malignant ureteral obstruction or injury. Urology.

[5] European Association of Urology (2024). EAU guidelines on urological trauma. https://d56bochluxqnz.cloudfront.net/documents/full-guideline/EAU-Guidelines-on-Urological-Trauma-2024_2024-04-11-083052_vlwe.pdf.

[6] Stein R, Rubenwolf P, Ziesel C, Kamal MM, Thüroff JW (2013). Psoas hitch and Boari flap ureteroneocystostomy. BJU Int.

[7] White C, Stifelman M (2020). Ureteral reimplantation, psoas hitch, and Boari flap. J Endourol.

[8] Mauck RJ, Hudak SJ, Terlecki RP, Morey AF (2011). Central role of Boari bladder flap and downward nephropexy in upper ureteral reconstruction. J Urol.

[9] Zhong MZ, Huang WN, Huang GX, Zhang EP, Gan L (2022). Long-term results of extended Boari flap technique for management of complete ureteral avulsion: a case report. World J Clin Cases.

[10] Kromann B, Steven K, Hald T, Olsson C (1986). The use of the Boari-flap and psoas-bladder hitch technique in the repair of a high ureteric lesion. A case report. Scand J Urol Nephrol.

[11] Knight RB, Hudak SJ, Morey AF (2013). Strategies for open reconstruction of upper ureteral strictures. Urol Clin North Am.

[12] Harada N, Tanimura M, Fukuyama K, Asakura T, Morimoto Y, Hattori H (1964). Surgical management of a long ureteral defect: advancement of the ureter by descent of the kidney. J Urol.

